# 2-(3-Meth­oxy­phen­yl)-1,3-dihydro-1,3,2-benzodiaza­borole

**DOI:** 10.1107/S1600536812028437

**Published:** 2012-06-30

**Authors:** Ross S. Robinson, Siphamandla Sithebe, Matthew P. Akerman

**Affiliations:** a School of Chemistry and Physics, University of KwaZulu-Natal, Private Bag X01, Scottsville, Pietermaritzburg 3209, South Africa

## Abstract

The title compound, C_13_H_13_BN_2_O, is one in a series of 1,3,2-benzodiaza­boroles featuring a 2-meth­oxy­phenyl substitution at the 2-position in the nitro­gen–boron heterocyle. The dihedral angle between the mean planes of the benzodiaza­borole and 2-meth­oxy­phenyl ring systems is 21.5 (1)°. There is an inter­molecular hydrogen bond between one of the NH groups and the meth­oxy O atom. This hydrogen bond leads to an infinite hydrogen-bonded chain colinear with the *a* axis.

## Related literature

For the synthesis of the title compound, see: Sithebe *et al.* (2011 ▶); Weber *et al.* (2009 ▶, 2011 ▶). For related derivatives as well as their photoluminiscence studies, see: Weber *et al.* (2010 ▶); Maruyama & Kawanishi (2002 ▶). For structures of related compounds, see: Slabber *et al.* (2011 ▶); Akerman *et al.* (2011 ▶). For applications of 1,3,2-diaza­borolyl compounds, see: Schwedler *et al.* (2011 ▶).
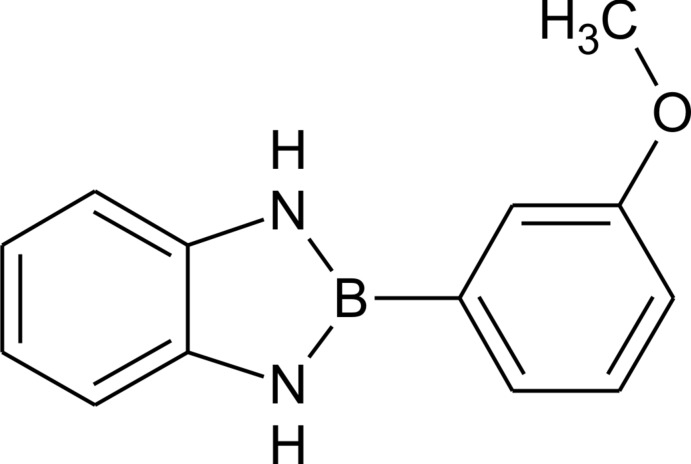


## Experimental

### 

#### Crystal data

C_13_H_13_BN_2_O
*M* *_r_* = 224.06
Orthorhombic, 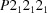

*a* = 7.549 (5) Å
*b* = 12.230 (5) Å
*c* = 12.308 (5) Å
*V* = 1136.3 (10) Å^3^
*Z* = 4
Mo *K*α radiation
μ = 0.08 mm^−1^
*T* = 110 K
0.50 × 0.40 × 0.40 mm

#### Data collection

Oxford Diffraction Xcalibur 2 CCD diffractometer
Absorption correction: multi-scan (Blessing, 1995 ▶) *T*_min_ = 0.960, *T*_max_ = 0.968
11586 measured reflections
2125 independent reflections
1939 reflections with *I* > 2σ(*I*)
*R*_int_ = 0.031

#### Refinement

*R*[*F* ^2^ > 2σ(*F* ^2^)] = 0.034
*wR*(*F* ^2^) = 0.094
*S* = 1.05
2125 reflections
164 parameters
H atoms treated by a mixture of independent and constrained refinement
Δρ_max_ = 0.32 e Å^−3^
Δρ_min_ = −0.20 e Å^−3^

### 

Data collection: *CrysAlis CCD* (Oxford Diffraction, 2008 ▶); cell refinement: *CrysAlis CCD*; data reduction: *CrysAlis RED* (Oxford Diffraction, 2008 ▶); program(s) used to solve structure: *SHELXS97* (Sheldrick, 2008 ▶); program(s) used to refine structure: *SHELXL97* (Sheldrick, 2008 ▶); molecular graphics: *Mercury* (Macrae *et al.*, 2006 ▶) and *POV-RAY* (Cason *et al.*, 2002 ▶); software used to prepare material for publication: *publCIF* (Westrip, 2010 ▶).

## Supplementary Material

Crystal structure: contains datablock(s) I, global. DOI: 10.1107/S1600536812028437/nk2170sup1.cif

Structure factors: contains datablock(s) I. DOI: 10.1107/S1600536812028437/nk2170Isup2.hkl

Supplementary material file. DOI: 10.1107/S1600536812028437/nk2170Isup3.cml

Additional supplementary materials:  crystallographic information; 3D view; checkCIF report

## Figures and Tables

**Table 1:** Hydrogen-bond geometry (Å, °)

*D*—H⋯*A*	*D*—H	H⋯*A*	*D*⋯*A*	*D*—H⋯*A*
N2—H102⋯O001^i^	0.89 (2)	2.40 (2)	3.201 (2)	151 (2)

Symmetry code: (i) 
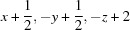
.

**Fig. 1.**
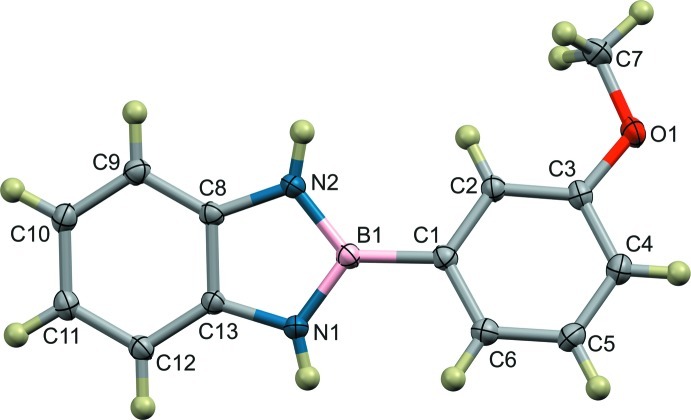
Displacement ellipsoid plot of (1) at the 50% probability level.

**Fig. 2.**
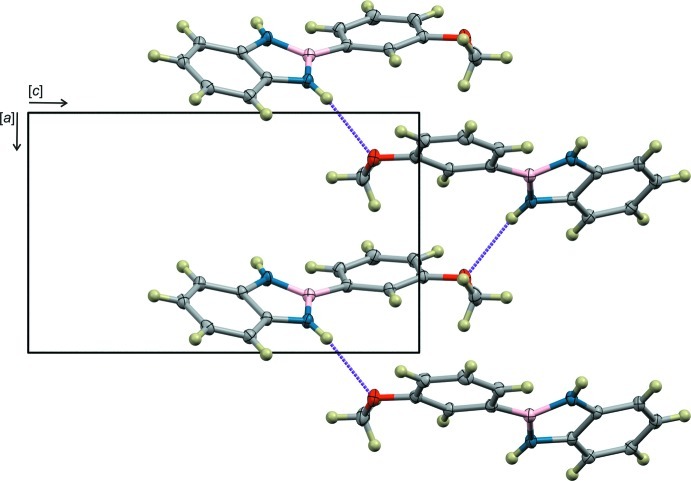
Hydrogen bonding interactions in (1), shown as dashed lines, viewed down the b-axis.

## supplementary crystallographic information

### Comment

Molecular compounds functionalized with 1,3,2-diazaborolyl groups have received considerable attention and have been investigated for their optical, electronic and ion sensing abilities, making them possible candidates for use in advanced material science (Schwedler *et al.*,2011). Rapid developments in the chemistry of 1,3,2-diazaborolyl containing compounds, due to their photoluminescence characteristics and unusual stability, have been observed in recent years. Unlike most triarylborane compounds which require dimesitylborolyl moieties for the enhancement of their stability, 2-arylbenzo-1,3,2-diazaborole compounds have been reported to be water and air stable without any additional dimesityl groups (Weber *et al.*, 2009). To gain insight into the intriguing characteristics exhibited by these compounds, we (Sithebe *et al.*, 2011) and other researchers (Maruyama *et al.*, 2002 and Weber *et al.* 2011) have directed our reseach focus towards the investigation of the photophysical studies as well as the determination of the crystal structures of 1,3,2-benzodiazaborolyl compounds.

The molecule features a 1,3,2-benzodiazaborolyl backbone with a five-membered diazaborole ring substituted with hydrogen atoms at the 1- and 3-positions, and a 3-methoxyphenyl ring at the 2-position. The 1,3,2-benzodiazaborolyl backbone of the molecule is essentially planar, however, the 3-methoxyphenyl ring at the 2-position, is rotated out of plane with a dihedral angle of 21.5 (1)°. The two N—B bonds are approximately equal (averaged to 1.433 (2) Å). The N1—B—N2 bond angle is 105.2 (1)°, the N1—B—C1 and N2—B—C1 bond angles are slighly different, measuring 125.4 (1)° and 129.3 (1)°, respectively (refer to Figure 1 for the atom numbering scheme). These bond lengths and angles compare favourably to those of previously reported diazaborolyl systems (Weber *et al.*, 2009). The molecules are linked through hydrogen bonding forming infinite, one-dimensional chains co-linear with the *a*-axis (Figure 2). The amine NH acts as the hydrogen bond donor and the etheryl oxygen atom the H-bond acceptor. The hydrogen bond lengths and bond angles are summarized in Table 1.

### Experimental

3-Methoxyphenylboronic acid (1.00 g, 5.18 mmol) and *o-*phenylenediamine (0.56 g, 5.18 mmol) were dissolved in toluene (80 ml) in a two neck flask equipped with a Dean and Stark Apparatus, magnetic stirrer bar and reflux condenser. The mixture was heated under reflux overnight and the solvent was removed *in vacuo*, affording 2-{3-methoxyphenyl}benzo-1,3,2-diazaborole as an off-white solid. The desired product was purified using a flash column and radial chromatography using Hexane: Ethyl acetate (8:2) as the eluent. Crystals suitable for X-ray difraction were grown by slow evaporation of a *n*-hexane:dicloromethane (6:4) solution.

### Refinement

All non-hydrogen atoms were located in the difference Fourier map and refined anisotropically. The positions of all hydrogen atoms were calculated using the standard riding model of *SHELXL97.* with C—H(aromatic) distances of 0.93 Å and *U*_iso_ = 1.2 *U*_eq_, and *C*—*H*(methyl) distances of 0.96 Å and *U*_iso_ = 1.5 *U*_eq_. The amine hydrogen atoms were located in the difference Fourier map and allowed to refine isotropically. In the absence of significant anomalous scattering, Friedel pairs were merged.

### Crystal data

**Table:**

C_13_H_13_BN_2_O	*F*(000) = 472
*M**_r_* = 224.06	*D*_x_ = 1.310 Mg m^−^^3^
Orthorhombic, *P*2_1_2_1_2_1_	Mo *K*α radiation, λ = 0.71073 Å
Hall symbol: P 2ac 2ab	Cell parameters from 1939 reflections
*a* = 7.549 (5) Å	θ = 3.2–32.1°
*b* = 12.230 (5) Å	µ = 0.08 mm^−^^1^
*c* = 12.308 (5) Å	*T* = 110 K
*V* = 1136.3 (10) Å^3^	Needle, colourless
*Z* = 4	0.50 × 0.40 × 0.40 mm

### Data collection

**Table:**

Oxford Diffraction Xcalibur 2 CCD diffractometer	2125 independent reflections
Radiation source: fine-focus sealed tube	1939 reflections with *I* > 2σ(*I*)
Graphite monochromator	*R*_int_ = 0.031
ω scans at fixed θ angles	θ_max_ = 32.1°, θ_min_ = 3.2°
Absorption correction: multi-scan (Blessing, 1995)	*h* = −11→7
*T*_min_ = 0.960, *T*_max_ = 0.968	*k* = −17→18
11586 measured reflections	*l* = −18→18

### Refinement

**Table:**

Refinement on *F*^2^	Secondary atom site location: difference Fourier map
Least-squares matrix: full	Hydrogen site location: inferred from neighbouring sites
*R*[*F*^2^ > 2σ(*F*^2^)] = 0.034	H atoms treated by a mixture of independent and constrained refinement
*wR*(*F*^2^) = 0.094	*w* = 1/[σ^2^(*F*_o_^2^) + (0.0725*P*)^2^] where *P* = (*F*_o_^2^ + 2*F*_c_^2^)/3
*S* = 1.05	(Δ/σ)_max_ < 0.001
2125 reflections	Δρ_max_ = 0.32 e Å^−^^3^
164 parameters	Δρ_min_ = −0.20 e Å^−^^3^
0 restraints	Extinction correction: *SHELXL97* (Sheldrick, 2008), Fc^*^=kFc[1+0.001xFc^2^λ^3^/sin(2θ)]^-1/4^
Primary atom site location: structure-invariant direct methods	Extinction coefficient: 0.058 (6)

### Special details

**Table:**

Geometry. All s.u.'s (except the s.u. in the dihedral angle between two l.s. planes) are estimated using the full covariance matrix. The cell s.u.'s are taken into account individually in the estimation of s.u.'s in distances, angles and torsion angles; correlations between s.u.'s in cell parameters are only used when they are defined by crystal symmetry. An approximate (isotropic) treatment of cell s.u.'s is used for estimating s.u.'s involving l.s. planes.
Refinement. Refinement of *F*^2^ against ALL reflections. The weighted *R*-factor *wR* and goodness of fit *S* are based on *F*^2^, conventional *R*-factors *R* are based on *F*, with *F* set to zero for negative *F*^2^. The threshold expression of *F*^2^ > 2σ(*F*^2^) is used only for calculating *R*-factors(gt) *etc*. and is not relevant to the choice of reflections for refinement. *R*-factors based on *F*^2^ are statistically about twice as large as those based on *F*, and *R*- factors based on ALL data will be even larger.

### Fractional atomic coordinates and isotropic or equivalent isotropic displacement parameters (Å^2^)

**Table:**

	*x*	*y*	*z*	*U*_iso_*/*U*_eq_	
O001	0.68887 (13)	0.34747 (7)	1.11670 (7)	0.0238 (2)	
N2	0.86812 (15)	0.17066 (8)	0.71418 (8)	0.0184 (2)	
N1	0.69146 (14)	0.27984 (8)	0.61068 (8)	0.0187 (2)	
C8	0.85853 (16)	0.12575 (9)	0.61012 (9)	0.0171 (2)	
C2	0.73726 (16)	0.30830 (10)	0.92377 (10)	0.0179 (2)	
H2	0.7848	0.2380	0.9388	0.022*	
C6	0.65474 (16)	0.44957 (10)	0.79625 (10)	0.0217 (2)	
H6	0.6449	0.4756	0.7238	0.026*	
C11	0.78716 (17)	0.07371 (10)	0.39528 (10)	0.0208 (2)	
H11	0.7634	0.0544	0.3219	0.025*	
C13	0.74943 (16)	0.19384 (9)	0.54613 (9)	0.0168 (2)	
C12	0.71345 (16)	0.16880 (10)	0.43846 (9)	0.0194 (2)	
H12	0.6407	0.2151	0.3954	0.023*	
C1	0.72155 (16)	0.34445 (10)	0.81557 (9)	0.0181 (2)	
C9	0.93295 (17)	0.03188 (10)	0.56632 (11)	0.0203 (2)	
H9	1.0075	−0.0139	0.6087	0.024*	
C4	0.61570 (17)	0.47951 (10)	0.98828 (10)	0.0228 (3)	
H4	0.5789	0.5249	1.0467	0.027*	
C3	0.68309 (17)	0.37557 (10)	1.00871 (10)	0.0193 (2)	
C5	0.60273 (18)	0.51624 (10)	0.88201 (11)	0.0241 (3)	
H5	0.5580	0.5874	0.8677	0.029*	
C10	0.89523 (17)	0.00661 (10)	0.45839 (10)	0.0213 (2)	
H10	0.9442	−0.0577	0.4272	0.026*	
C7	0.7662 (2)	0.24408 (11)	1.14365 (11)	0.0296 (3)	
H7A	0.6947	0.1852	1.1122	0.044*	
H7B	0.7700	0.2358	1.2228	0.044*	
H7C	0.8868	0.2405	1.1144	0.044*	
B1	0.76235 (18)	0.26817 (10)	0.71788 (10)	0.0179 (2)	
H102	0.940 (3)	0.1411 (15)	0.7630 (17)	0.042 (5)*	
H101	0.625 (3)	0.3271 (17)	0.5846 (18)	0.050 (6)*	

### Atomic displacement parameters (Å^2^)

**Table:**

	*U*^11^	*U*^22^	*U*^33^	*U*^12^	*U*^13^	*U*^23^
O001	0.0320 (5)	0.0242 (4)	0.0153 (4)	0.0015 (4)	0.0037 (4)	−0.0013 (3)
N2	0.0218 (5)	0.0184 (4)	0.0150 (4)	0.0016 (4)	−0.0021 (4)	0.0006 (3)
N1	0.0233 (5)	0.0172 (4)	0.0156 (4)	0.0040 (4)	−0.0009 (4)	0.0001 (4)
C8	0.0195 (5)	0.0166 (5)	0.0152 (5)	−0.0009 (4)	0.0005 (4)	0.0018 (4)
C2	0.0197 (5)	0.0175 (5)	0.0166 (5)	−0.0002 (4)	0.0004 (4)	−0.0011 (4)
C6	0.0237 (6)	0.0211 (5)	0.0202 (5)	0.0015 (5)	−0.0015 (5)	0.0007 (4)
C11	0.0239 (6)	0.0214 (5)	0.0172 (5)	−0.0029 (5)	0.0019 (5)	−0.0017 (4)
C13	0.0196 (5)	0.0158 (4)	0.0150 (5)	0.0004 (4)	0.0013 (4)	0.0009 (4)
C12	0.0224 (5)	0.0207 (5)	0.0151 (5)	0.0000 (4)	−0.0003 (4)	0.0006 (4)
C1	0.0188 (5)	0.0185 (5)	0.0170 (5)	−0.0006 (4)	−0.0008 (4)	−0.0006 (4)
C9	0.0219 (5)	0.0182 (5)	0.0207 (5)	0.0018 (4)	0.0015 (5)	0.0018 (4)
C4	0.0243 (6)	0.0220 (6)	0.0221 (6)	0.0024 (5)	0.0021 (5)	−0.0036 (4)
C3	0.0202 (5)	0.0210 (5)	0.0166 (5)	−0.0022 (4)	0.0010 (4)	−0.0020 (4)
C5	0.0265 (6)	0.0194 (5)	0.0265 (6)	0.0047 (5)	−0.0011 (5)	−0.0010 (5)
C10	0.0242 (6)	0.0180 (5)	0.0219 (5)	0.0004 (5)	0.0041 (5)	−0.0015 (4)
C7	0.0382 (7)	0.0313 (7)	0.0195 (5)	0.0066 (6)	0.0031 (5)	0.0052 (5)
B1	0.0202 (5)	0.0178 (5)	0.0157 (5)	−0.0005 (5)	0.0001 (5)	0.0003 (4)

### Geometric parameters (Å, º)

**Table:**

O001—C3	1.3735 (15)	C11—C10	1.3937 (18)
O001—C7	1.4316 (17)	C11—C12	1.3945 (17)
N2—C8	1.3954 (15)	C11—H11	0.9500
N2—B1	1.4359 (17)	C13—C12	1.3870 (16)
N2—H102	0.89 (2)	C12—H12	0.9500
N1—C13	1.3888 (15)	C1—B1	1.5527 (18)
N1—B1	1.4309 (17)	C9—C10	1.3933 (19)
N1—H101	0.83 (2)	C9—H9	0.9500
C8—C9	1.3872 (17)	C4—C5	1.3864 (19)
C8—C13	1.4115 (16)	C4—C3	1.3921 (18)
C2—C3	1.3918 (16)	C4—H4	0.9500
C2—C1	1.4081 (17)	C5—H5	0.9500
C2—H2	0.9500	C10—H10	0.9500
C6—C5	1.3903 (18)	C7—H7A	0.9800
C6—C1	1.4013 (17)	C7—H7B	0.9800
C6—H6	0.9500	C7—H7C	0.9800
			
C3—O001—C7	117.27 (10)	C6—C1—B1	119.42 (11)
C8—N2—B1	109.10 (10)	C2—C1—B1	121.80 (11)
C8—N2—H102	119.5 (12)	C8—C9—C10	118.12 (12)
B1—N2—H102	131.1 (12)	C8—C9—H9	120.9
C13—N1—B1	109.52 (10)	C10—C9—H9	120.9
C13—N1—H101	119.8 (15)	C5—C4—C3	119.48 (12)
B1—N1—H101	130.7 (15)	C5—C4—H4	120.3
C9—C8—N2	131.41 (11)	C3—C4—H4	120.3
C9—C8—C13	120.51 (11)	O001—C3—C2	124.73 (11)
N2—C8—C13	108.07 (10)	O001—C3—C4	114.51 (11)
C3—C2—C1	120.01 (11)	C2—C3—C4	120.76 (12)
C3—C2—H2	120.0	C4—C5—C6	120.41 (12)
C1—C2—H2	120.0	C4—C5—H5	119.8
C5—C6—C1	120.72 (12)	C6—C5—H5	119.8
C5—C6—H6	119.6	C9—C10—C11	121.36 (12)
C1—C6—H6	119.6	C9—C10—H10	119.3
C10—C11—C12	120.81 (12)	C11—C10—H10	119.3
C10—C11—H11	119.6	O001—C7—H7A	109.5
C12—C11—H11	119.6	O001—C7—H7B	109.5
C12—C13—N1	130.70 (11)	H7A—C7—H7B	109.5
C12—C13—C8	121.14 (11)	O001—C7—H7C	109.5
N1—C13—C8	108.15 (10)	H7A—C7—H7C	109.5
C13—C12—C11	118.05 (12)	H7B—C7—H7C	109.5
C13—C12—H12	121.0	N1—B1—N2	105.16 (10)
C11—C12—H12	121.0	N1—B1—C1	125.44 (11)
C6—C1—C2	118.61 (11)	N2—B1—C1	129.33 (11)
			
B1—N2—C8—C9	−178.52 (13)	C7—O001—C3—C4	−176.53 (12)
B1—N2—C8—C13	0.68 (13)	C1—C2—C3—O001	178.47 (12)
B1—N1—C13—C12	178.50 (13)	C1—C2—C3—C4	−1.02 (18)
B1—N1—C13—C8	−0.17 (14)	C5—C4—C3—O001	−179.61 (12)
C9—C8—C13—C12	0.17 (18)	C5—C4—C3—C2	−0.07 (19)
N2—C8—C13—C12	−179.14 (11)	C3—C4—C5—C6	0.7 (2)
C9—C8—C13—N1	178.99 (10)	C1—C6—C5—C4	−0.2 (2)
N2—C8—C13—N1	−0.32 (13)	C8—C9—C10—C11	0.53 (18)
N1—C13—C12—C11	−178.02 (12)	C12—C11—C10—C9	0.14 (19)
C8—C13—C12—C11	0.50 (18)	C13—N1—B1—N2	0.57 (13)
C10—C11—C12—C13	−0.65 (18)	C13—N1—B1—C1	−176.89 (11)
C5—C6—C1—C2	−0.88 (19)	C8—N2—B1—N1	−0.76 (13)
C5—C6—C1—B1	174.46 (12)	C8—N2—B1—C1	176.55 (12)
C3—C2—C1—C6	1.48 (18)	C6—C1—B1—N1	−19.86 (19)
C3—C2—C1—B1	−173.75 (12)	C2—C1—B1—N1	155.33 (12)
N2—C8—C9—C10	178.44 (12)	C6—C1—B1—N2	163.32 (12)
C13—C8—C9—C10	−0.68 (17)	C2—C1—B1—N2	−21.5 (2)
C7—O001—C3—C2	3.95 (18)		

### Hydrogen-bond geometry (Å, º)

**Table:**

*D*—H···*A*	*D*—H	H···*A*	*D*···*A*	*D*—H···*A*
N2—H102···O001^i^	0.89 (2)	2.40 (2)	3.201 (2)	151 (2)

Symmetry code: (i) *x*+1/2, −*y*+1/2, −*z*+2.
